# Multi‐Scale Habitat Suitability and Spatial Distribution of the European Green Toad

**DOI:** 10.1002/ece3.73984

**Published:** 2026-07-07

**Authors:** Ali Erdem Özçelik, Rıdvan Ertuğrul Yıldırım, Abdullah Altunışık

**Affiliations:** ^1^ Department of Landscape Architecture, Faculty of Engineering and Architecture Recep Tayyip Erdogan University Rize Türkiye; ^2^ Department of Geomatics, Faculty of Engineering Ondokuz Mayis University Samsun Türkiye; ^3^ Biology Department, Faculty of Arts and Sciences Recep Tayyip Erdogan University Rize Türkiye

**Keywords:** amphibian conservation, analytic hierarchy process, *Bufotes viridis*, ecological corridors, GIS‐based modeling, habitat suitability, spatial distribution, Türkiye

## Abstract

Amphibians are among the most threatened vertebrates globally, primarily because of habitat loss, pollution, climate change, and landscape fragmentation. This study aimed to model and map the potential spatial distribution and ecological suitability of the European green toad ( *Bufotes viridis sensu lato* ) across the Mediterranean Basin of Türkiye to inform conservation and habitat management strategies. A multi‐criteria decision‐making approach integrated with Geographic Information Systems (GIS) was applied. The Analytic Hierarchy Process (AHP) was used to assign weights to nine environmental variables: slope, aspect, altitude, canopy cover, temperature, land cover, and distances to roads, urban centers, and freshwater. The weighted layers were combined to produce a composite habitat suitability map. The model accuracy was validated using 33 georeferenced field sampling points from Adana Province. Model outputs indicated that approximately 67% of the study area was classified as high or very high suitability for *B. viridis* , while 95.36% of the study area fell within the moderate, high, or very high suitability classes. Proximity to freshwater (weight = 0.352) and temperature (weight = 0.186) were identified as the most influential predictors. Spatial analysis revealed that suitable habitats are primarily concentrated near freshwater systems and low‐elevation zones, highlighting the importance of riparian and agricultural landscapes as key breeding and dispersal areas. The integrative AHP–GIS framework successfully identified critical breeding habitats, ecological corridors, and priority conservation zones for *B. viridis* . Our findings provide a spatially explicit framework that supports biodiversity conservation and sustainable landscape management in Mediterranean ecosystems, offering a replicable model for amphibian species in other biodiversity‐rich but data‐deficient regions.

## Graphical abstract

We modeled the multi‐scale spatial distribution and habitat suitability of the European green toad ( *Bufotes viridis* ) in Mediterranean Türkiye using an AHP–GIS framework integrating key environmental variables. The model identified proximity to freshwater and temperature as the strongest predictors, with ~67% of the area classified as highly suitable, primarily in lowland and riparian landscapes. These results reveal ecologically important breeding habitats and connectivity corridors, providing a spatial basis for conservation prioritization under increasing anthropogenic pressure.

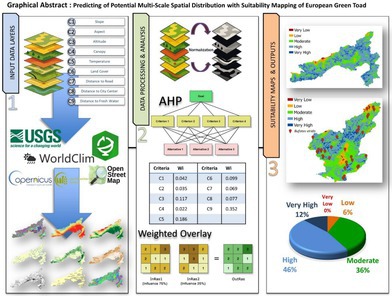

## 1 Introduction

The spatial scale at which landscape features most strongly influence species populations is crucial in determining landscape connectivity. As a scale‐dependent property, landscape connectivity regulates species movement and dispersal within heterogeneous land use and land cover (LULC) mosaics. There is a close interplay between connectivity, species' geographical ranges, and population dynamics (Gengler et al. 2024). Therefore, maintaining viable amphibian populations in human‐modified environments requires regional evaluations of landscape connectivity to identify ecological corridors and dispersal barriers, which can inform the development of effective conservation and management strategies (Caballero‐Díaz et al. 2025). Although various analytical methods have been developed to determine biologically meaningful spatial scales, identifying the primary ecological factors that shape species' spatial distributions remains an ongoing challenge. Moreover, these ecological drivers may vary across both geographic regions and time periods, yet comprehensive studies examining spatiotemporal variation in species occurrence are still limited (Pease 2024). Evaluating the capacity of a habitat to support a viable population over ecologically relevant time frames provides an effective approach for assessing habitat quality and predicting potential distribution patterns (Edosa and Erena 2024). Therefore, identifying geographically suitable areas for species persistence is a fundamental objective in wildlife conservation, particularly for environmentally sensitive amphibian species.

Amphibians are experiencing severe global declines, largely because of habitat loss, land‐use change, pollution, emerging diseases, and climate change (Campbell et al. 2024; Chen et al. 2025; Altunışık 2026). Amphibians are highly sensitive to environmental alterations and therefore represent effective bioindicators of ecosystem change because of their permeable skin and reliance on both aquatic and terrestrial habitats (Han et al. 2024; Wu et al. 2023). Integrating occurrence records with habitat preference data from the literature is essential to delineate observed ranges and potential distributional areas that may serve as future conservation priorities (Ficetola et al. 2014; Préau et al. 2019).

The European green toad, *Bufotes viridis* sensu lato, is broadly distributed across Europe and Western Asia and is often considered ecologically flexible (Dufresnes et al. 2019; Stöck et al. 2006). It occurs in a wide range of open and semi‐open habitats, including agricultural mosaics, ruderal sites, urban peripheries, and temporary or permanent aquatic habitats required for reproduction (Edwards et al. 2025; Luo et al. 2021). Despite this broad ecological tolerance, regional populations often display distinct habitat associations, indicating that local distribution is driven by a combination of environmental variables, such as land cover, hydrological availability, elevation, and landscape configuration (Caballero‐Díaz et al. 2025; Sillero et al. 2014). However, detailed and regionally specific habitat suitability maps that integrate empirical records with environmental drivers remain scarce (Pottier et al. 2025; Sun et al. 2025). In this study, we combined published information on habitat preferences with national occurrence records to evaluate the environmental correlates of *B. viridis* presence and predict its potential distribution. We hypothesized that the local distribution of *B. viridis* is shaped by hydrological availability (e.g., presence of breeding sites and proximity to water), land use/land cover composition (e.g., presence of open and semi‐open habitats), and topographic constraints (e.g., elevation and slope). When analyzed at multiple spatial scales and integrated with literature‐based ecological requirements, these drivers are expected to yield consistent spatial patterns that can be modeled using GIS‐based multi‐criteria approaches (Edosa and Erena 2024; Sivrikaya et al. 2022). This study aimed to provide the first spatially explicit assessment of the current and potential distribution of *B. viridis* in the Mediterranean Basin of Türkiye, offering a novel perspective on the species' habitat ecology and conservation needs. By synthesizing habitat preferences reported in the literature with regional occurrence data, we assessed the relative contribution of environmental variables to species distribution and produced predictive habitat suitability maps. These outputs not only highlight under‐surveyed areas of ecological potential but also provide practical guidance for conservation planning, such as the identification of critical breeding habitats, prioritization of monitoring sites, and development of habitat management strategies under ongoing land‐use and climate change pressures.

## 2 Methods

**FIGURE 1:**
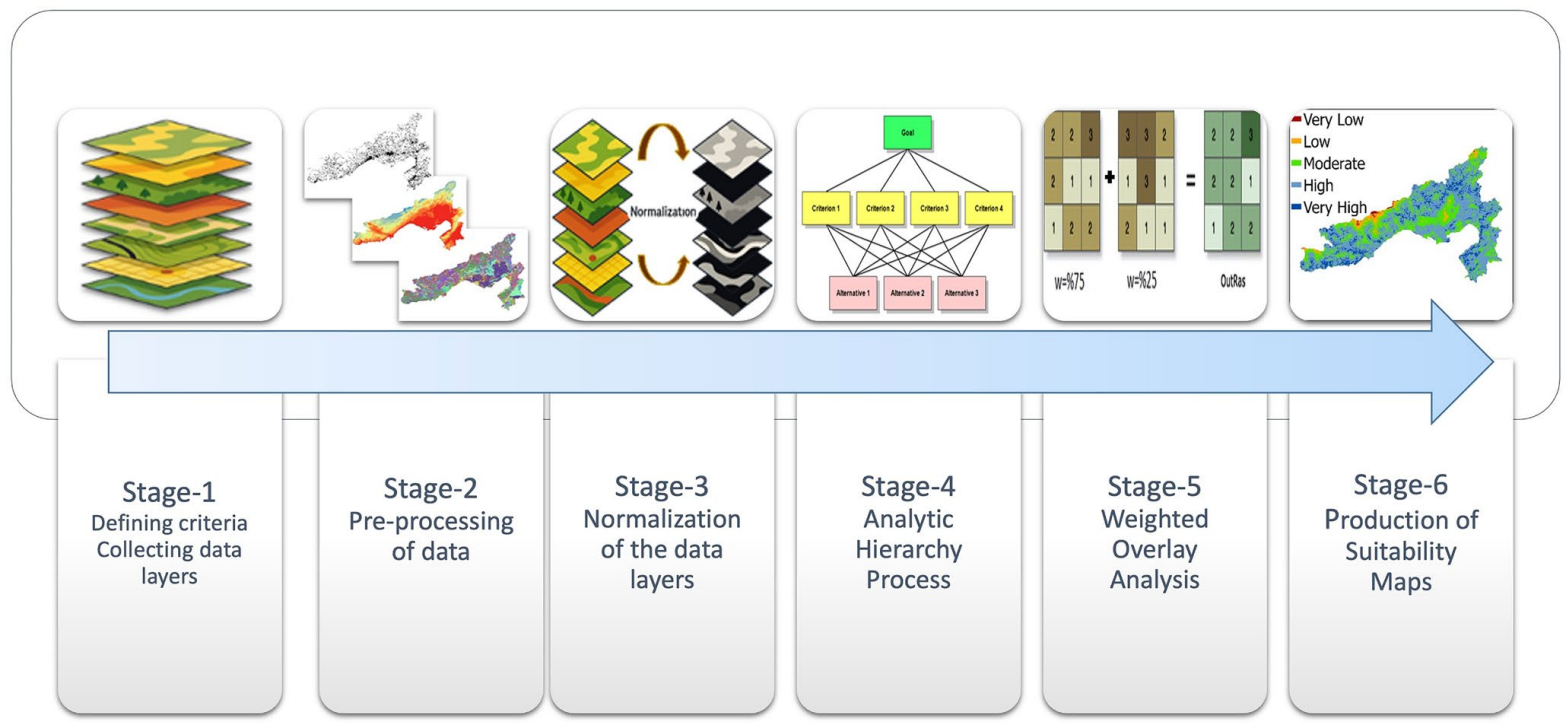
Data processing workflow.

To ensure consistency and comparability among all spatial layers, a comprehensive preprocessing workflow (Figure 1) was applied to both vector and raster data. The following steps were executed:

The workflow of the study consisted of six standardized stages (Figure 1). The process commenced with the definition of the evaluation criteria and the collection of the relevant data layers (Stage 1). All input datasets were clipped to the geographical boundaries of the study area to ensure spatial uniformity and focus the analysis on relevant regions. Once the necessary datasets were obtained, they were subjected to a pre‐processing stage (Stage 2), in which the raw data were prepared for subsequent analyses through procedures such as format harmonization, spatial alignment, and resolution adjustment.

Vector datasets (roads, city centers, freshwater bodies) were converted into raster format with a common spatial resolution to enable raster‐based analysis. Euclidean distance rasters were generated for proximity‐based criteria (roads, city centers, freshwater) using Euclidean Distance tools. These rasters were then reclassified into ordinal suitability classes. Following this, all data layers were normalized (Stage 3) to a common scale, ensuring comparability among heterogeneous variables and minimizing bias introduced by differences in measurement units. Continuous variables (slope, aspect, altitude, temperature) were reclassified into suitability categories based on ecological thresholds and expert‐defined intervals. The AHP (Stage 4) was then applied in order to derive the relative importance of each criterion, based on structured pairwise comparisons and consistency assessments. All raster layers were normalized to a [0–1] scale using min‐max normalization to allow for uniform contribution in the weighted overlay. In the next step, a weighted overlay analysis (Stage 5) was conducted, integrating the criteria weights with the normalized data layers to generate a composite suitability index. Output raster layers were reviewed for: Spatial alignment (projection and resolution), Absence of missing data, Logical consistency of classification ranges. This workflow ensured that all input data were harmonized and ready for integration into the suitability model. All raster layers were rescaled to a common scale of 0 to 1 using min‐max normalization. This process is required for the integration of diverse criteria within a unified analytical framework. The standardized preprocessing ensured that each layer contributed equitably to the final composite suitability index. Finally, suitability maps (Stage 6) were produced, providing a spatial representation of the outcomes and enabling the identification of areas with varying levels of suitability for the defined objective.

### 2.1 Study Area

**FIGURE 2:**
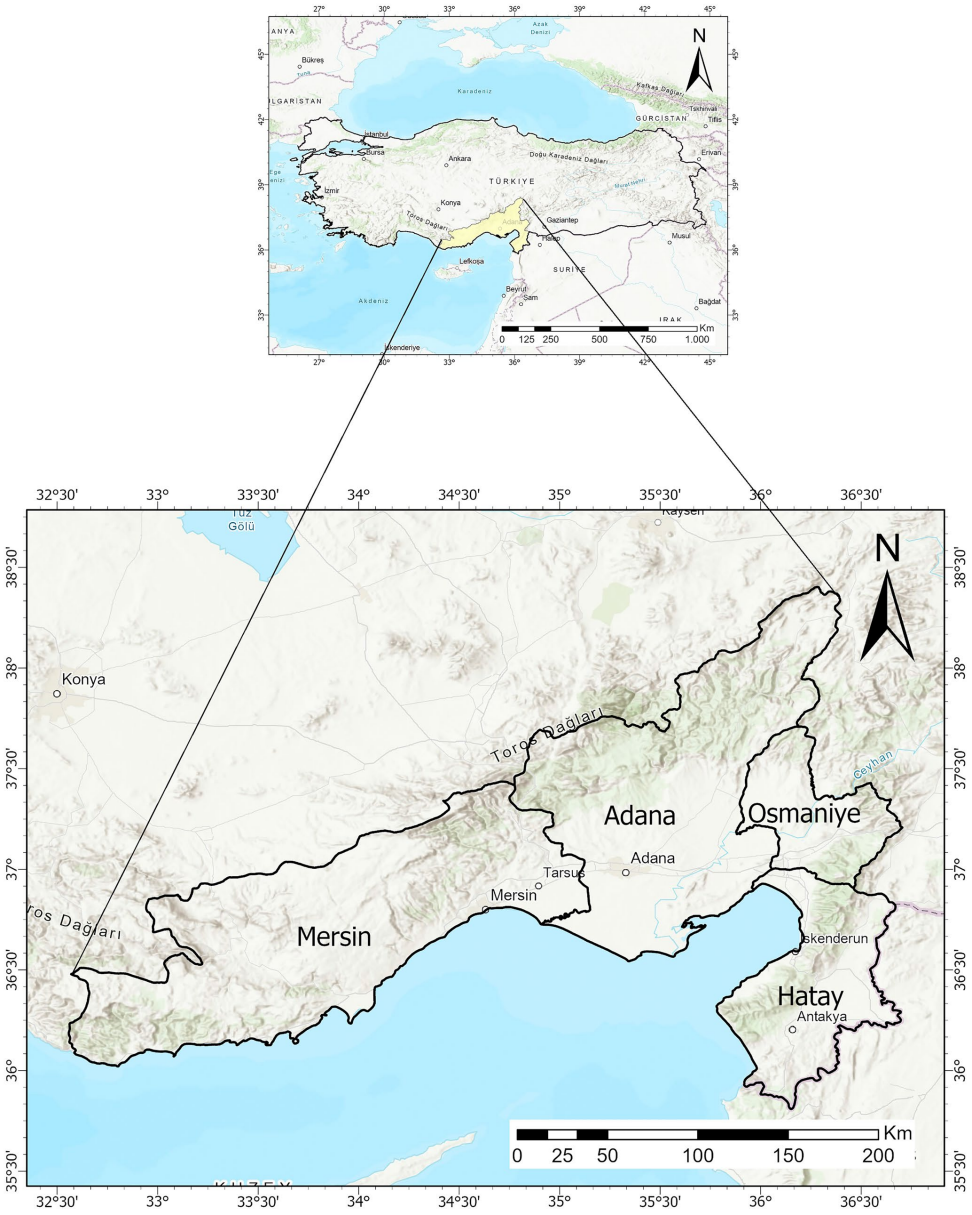
Geographical location of the study area.

The study area encompasses a broad geographic region within the Mediterranean Basin of Türkiye, including multiple provinces with ecological and topographical diversity (Figure 2). The selected region covers a variety of landscapes, such as coastal plains, forested uplands, mountainous zones, and semi‐arid inland areas. This heterogeneity in environmental conditions provides a robust basis for spatial modeling aimed at ecological‐suitability assessment.

While the spatial model was applied across the entire designated study area, field validation and comparative evaluation of the model outputs were performed using sampling points predominantly located within Adana Province. These points, selected based on available field data and expert‐verified locations, were overlaid on the final suitability map to evaluate the model's alignment with real‐world observations.

Adana was chosen for field‐based sampling due to its rich environmental gradients, ranging from sea level to mountainous terrain, and the presence of diverse ecosystems. This provided a representative subset of the broader region, facilitating meaningful interpretation of model results and allowing for preliminary validation of suitability classifications.

### 2.2 Studied Species

The European green toad ( *Bufotes viridis* sensu lato [Laurenti, 1768]) is a widely distributed amphibian complex occurring across Europe, western Asia, and parts of North Africa, with multiple cryptic lineages recognized within the group (Frost 2024; Dufresnes et al. 2019). In Türkiye, it is represented by the subspecies *B. v. sitibundus* (Pallas, 1769), which is widely distributed throughout the country and exhibits considerable ecological adaptability (Altunışık et al. 2021). It typically inhabits open and semi‐open landscapes such as grasslands, agricultural mosaics, ruderal areas, and urban environments, reflecting a high degree of ecological plasticity (Kuzmin 1999; Altunışık et al. 2021; IUCN SSC Amphibian Specialist Group 2023; Wells 2007). Reproduction typically occurs in shallow aquatic habitats such as temporary pools, ponds, ditches, and occasionally slow‐flowing waters, where fish absence and suitable hydroperiod are critical for larval development (Kuzmin 1999). In Türkiye, breeding generally takes place from March to June, peaking in April–May depending on local climatic conditions (Altunışık et al. 2021; Sarıkaya et al. 2017). Although the species shows ecological flexibility and can tolerate moderate disturbance and even saline conditions, reproductive success is strongly dependent on the availability and duration of suitable breeding waters (Altunışık et al. 2021; Wells 2007). As a thermophilic amphibian, *B. viridis* becomes active under relatively warm conditions (> 10°C), with optimal reproductive and larval development typically occurring under moderate to warm temperatures (approximately 15°C–28°C) (Kuzmin 1999; Wells 2007). The species occurs from sea level up to approximately 1500–2000 m a.s.l., although it is most abundant in lowland areas (IUCN SSC Amphibian Specialist Group 2023). Adult individuals generally exhibit strong site fidelity, with movements typically restricted to local spatial scales (< 1 km), while rare dispersal events may extend further depending on habitat connectivity. Empirical evidence from *B. viridis* populations in Türkiye supports predominantly short‐range movements (Altunışık et al. 2021), while broader amphibian movement ecology frameworks indicate that dispersal distances are generally limited and context‐dependent (Alex Smith and Green 2005; Wells 2007; IUCN SSC Amphibian Specialist Group 2023). Due to its sensitivity to habitat loss, fragmentation, and water pollution, *B. viridis* is widely regarded as a useful bioindicator species and a model organism for spatial ecology and conservation studies in human‐modified landscapes (Landler et al. 2023; Sillero et al. 2014). Its wide distribution, combined with local population declines and sensitivity to habitat degradation, makes it an appropriate focal species for evaluating habitat suitability patterns and supporting conservation planning in Mediterranean landscapes.

### 2.3 Criteria Selection and Data Sources

**FIGURE 3:**
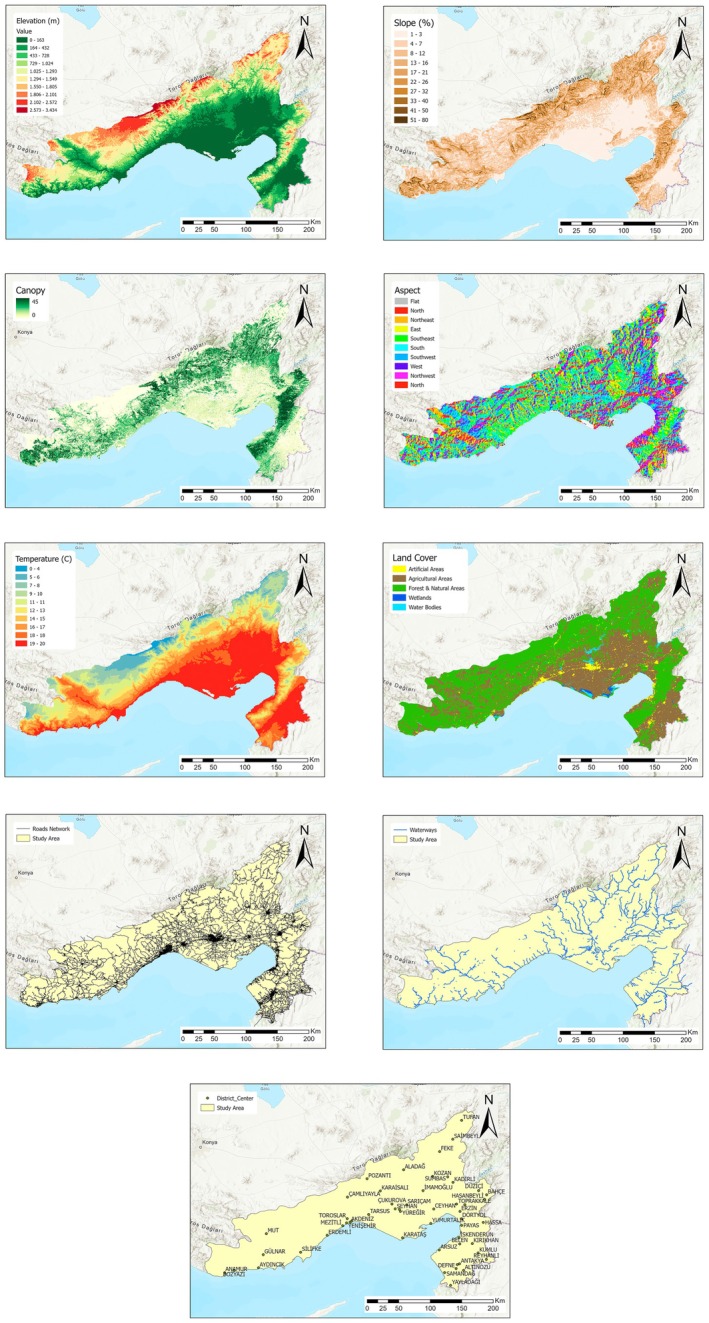
Spatial data layers of the criteria.

The dataset of nine spatial criteria and their corresponding geospatial layers (Figure 3) was selected based on ecological relevance, accessibility, and the availability of reliable data. Criterion selection was informed by a comprehensive review of the relevant literature and expert consultation involving three decision‐makers, including two specialists in biodiversity and amphibian ecology and one specialist in GIS‐based spatial analysis. The same experts subsequently participated in the AHP pairwise comparison process to determine the relative importance of the selected criteria. These criteria influence the ecological suitability of a given site and were incorporated into the suitability assessment framework as follows:

- Slope: Influences soil stability and vegetation growth.
- Aspect: Affects microclimate conditions and species distribution.
- Altitude: Correlates with temperature and habitat types.
- Canopy: Represents vegetation density and potential habitat coverage.
- Temperature: A key factor for species survival and distribution.
- Land Cover: Indicates the type of surface and its ecological potential.
- Distance to Road: Proximity to roads affects human disturbance and accessibility.
- Distance to City Center: Indicates anthropogenic pressure levels.
- Distance to Freshwater Sources: Crucial for biodiversity and habitat sustainability.

Each of these criteria was incorporated into the spatial model as individual layers and used to assess site suitability through a structured weighting and overlay process.

To support the multi‐criteria spatial analysis, openly accessible geospatial datasets were utilized for each of the selected criteria. The outline of the data sources and their corresponding sources (Table 1) was chosen based on their spatial resolution, update frequency, and compatibility with GIS‐based modeling.

**TABLE 1:** Data sources of the geospatial data layers.

Layer	Data source
Slope, aspect, altitude	USGS SRTM (Shuttle Radar Topography Mission)
Canopy	ESRI Living Atlas
Temperature	WorldClim 2.1 (1970–2000 Bioclimatic Variables)
Land cover/land use	CORINE (Copernicus Programme)
Distance to road	OpenStreetMap (OSM)
Distance to city center	OpenStreetMap (OSM)
Distance to freshwater	OpenStreetMap (OSM)

All spatial layers were acquired in raster or vector format and subsequently processed for consistency in resolution, projection (WGS84/UTM zone appropriate for the study area), and extent. To ensure spatial compatibility, all raster layers were generated and resampled to a common spatial resolution of 1000 m × 1000 m, corresponding to a pixel area of 1 km^2^.

### 2.4 Multi‐Criteria Decision Making Method: Analytic Hierarchy Process

The Analytic Hierarchy Process (AHP), developed by Thomas Saaty in the 1970s, is a structured technique used to organize and analyze complex decisions involving multiple criteria (Sipahi and Timor 2010; Yildirim and Sisman 2025). It facilitates decision‐making by breaking down a problem into sub‐problems, enabling systematic comparisons and prioritization through pairwise assessments (Ho and Ma 2018). Its simplicity and flexibility have led to its widespread application in fields such as environmental science, engineering, and strategic planning (Bernasconi et al. 2010; Ishizaka and Labib 2011). Furthermore, its ability to foster consensus among diverse stakeholders makes it particularly valuable in group decision‐making contexts because it streamlines the evaluation process and strengthens the justification of choices made (Baffoe 2019; Ishizaka and Labib 2011). The AHP remains a pivotal tool in multi‐criteria decision‐making, promoting systematic and transparent processes across a wide range of applications.

The AHP application in this study followed a structured sequence of steps to derive the criterion weights for the spatial‐suitability model. The process was executed as follows: the decision problem was structured hierarchically, with the overall goal of site suitability assessment placed at the top, evaluation criteria such as slope, temperature, and land cover in the middle, and the alternatives or spatial units at the bottom (Saaty 1990, 2004; Sipahi and Timor 2010). To determine the relative importance of these criteria, each was compared pairwise with the others using Saaty's 1–9 fundamental scale, which enables judgments ranging from “equally important” to “extremely more important” (Saaty 1990).

Subsequently, the pairwise comparison matrix was normalized by column, and the average of each row was calculated to obtain the relative weight, also referred to as the priority vector, of each criterion (Ho and Ma 2018). To ensure the consistency of these judgments, the Consistency Index (CI) and Consistency Ratio (CR) were computed, with a CR value below 0.10 accepted as an indication of satisfactory logical consistency (Saaty 1990, 2004). Finally, the derived weights were applied in a weighted linear combination to integrate the normalized spatial data layers into a composite suitability map (Bernasconi et al. 2010; Ishizaka and Labib 2011).

#### 2.4.1 Determination of Criteria and Sub‐Criteria

In this step, the decision‐making problem is clarified, followed by the determination of the associated criteria and sub‐criteria. These criteria embody the diverse factors that are expected to impact the overall evaluation process.

#### 2.4.2 Pairwise Comparison of Criteria

In this stage, experts or decision‐makers undertake pairwise evaluations of the criteria to determine their relative priorities, employing the Pairwise Comparison Method (PCM) (Equation 1). The outcome of this procedure is a comparison matrix, in which each element quantifies the relative dominance of one criterion over another, typically utilizing the 1–9 fundamental scale (Saaty 2004).

(1) APCM=1a12⋯a1n1a121⋯a2n⋮⋮⋮⋮1a1n1a2n⋯1

#### 2.4.3 Calculation of Criteria Weights

The pairwise comparison matrix is subsequently examined to obtain the weights of the criteria, achieved by normalizing the matrix and calculating the principal eigenvector corresponding to the maximum eigenvalue, which reflects the relative priority of each criterion (Equation 2) (Darko et al. 2019).

(2) $$\begin{array}{c} a_{\mathrm{ij}\prime }=\frac{a_{\mathrm{ij}}}{\sum_{i=1}^{n}a_{\mathrm{ij}}}\\ w_{i}=\frac{\sum_{i=1}^{n}a_{\mathrm{ij}\prime }}{n} \end{array}$$

where *a*_*ij′*_ is normalized weigths and *w*_*i*_ relative weights of each factor.

#### 2.4.4 Consistency Check

The relative weights are determined from the normalized eigenvector (*w*) associated with the maximum eigenvalue $\left(\lambda_{\max}\right)$. To assess the internal coherence of the judgments, the Consistency Ratio (CR) is calculated as the ratio of the Consistency Index (CI) to the Random Index (RI), where RI represents the average random consistency value dependent on the number of criteria (*n*) in the comparison. The Consistency Index (CI) is obtained according to Equation (3).

(3) $$\begin{array}{c} \lambda_{\max}=\frac{1}{n}\left(\frac{w_{1}^{\prime }}{w_{1}}+\frac{w_{2}^{\prime }}{w_{2}}+\cdots +\frac{w_{n}^{\prime }}{w_{n}}\right)\\ \mathrm{CI}=\frac{\lambda_{\max}-n}{n-1} \end{array}$$

Random Index (Saaty 2004);

**Table:** 

n	2	3	4	5	6	7	8	9	10
RI	0	0.52	0.49	1.11	1.25	1.35	1.40	1.45	1.49

(4) $$\mathrm{CR}=\frac{\mathrm{CI}}{\mathrm{RI}}$$

To evaluate the logical soundness of the pairwise comparison judgments, a consistency ratio (CR) is computed. When the CR value remains at or below the generally accepted threshold of 0.10, the judgments are regarded as consistent (Equation 4). Conversely, if the CR exceeds this limit, the reliability of the comparisons becomes questionable and the evaluation process may require revision (Saaty 2004).

### 2.5 Suitability Analysis

Following the preprocessing of spatial layers and the determination of criterion weights through AHP, a Weighted Overlay Analysis was conducted to generate the final ecological suitability map. Each normalized raster layer was multiplied by its corresponding AHP‐derived weight, and the resulting weighted raster data sets were summed to produce a composite suitability score for each pixel within the study area. The final suitability scores ranged between 0 (least suitable) and 1 (most suitable). The resulting continuous suitability map was normalized to a range of 0–1 and subsequently classified into five discrete suitability classes (Very Low, Low, Moderate, High, and Very High) using the equal interval classification method.

These classes allowed for intuitive interpretation of spatial patterns and facilitated the comparison of model results with known field observations. Example validation points from Adana province were overlaid to assess the spatial accuracy and ecological plausibility of the model outputs.

## 3 Results

The integration of weighted multi‐criteria inputs through GIS‐based overlay analysis resulted in a classified ecological suitability map, delineating zones ranging from Very Low to Very High suitability across the study area. This section presents the spatial patterns, distribution frequencies, and field‐level validation of model outputs.

### 3.1 Analytic Hierarchy Process

This stepwise methodology ensured that the weighting of criteria (Table 2) was grounded in both expert judgment and analytical rigor, aligning with best practices in multi‐criteria decision‐making for spatial planning. To determine the relative importance of each criterion in the site suitability analysis, the AHP was utilized. This structured multi‐criteria decision‐making approach incorporates expert judgment through pairwise comparisons to generate criterion weights based on their relative influence on ecological suitability. A pairwise comparison matrix was constructed based on expert assessments, and weights were calculated using the eigenvector method derived from the matrix. The consistency of judgments was evaluated using the Consistency Ratio (CR) to ensure logical coherence in the comparison process. In this study, the CR was calculated as 0.08376, confirming that the pairwise comparisons were both coherent and reliable.

**TABLE 2:** The weight of dataset of each data layer obtained from AHP process.

Criteria	AHP weight
Distance to fresh water	0.352
Temperature	0.186
Altitude	0.117
Land cover	0.099
Distance to city center	0.077
Distance to road	0.069
Slope	0.042
Aspect	0.035
Canopy	0.022

The calculated CR was 0.08376, which is well below the generally accepted threshold of 0.10. This indicates that the pairwise comparisons were consistent and the resulting weights are reliable for subsequent spatial modeling. These weights were subsequently applied in the weighted overlay analysis to integrate multiple criteria into a single suitability index.

### 3.2 Spatial Pattern Analysis for Spatial Distribution of Suitability Classes

**FIGURE 4:**
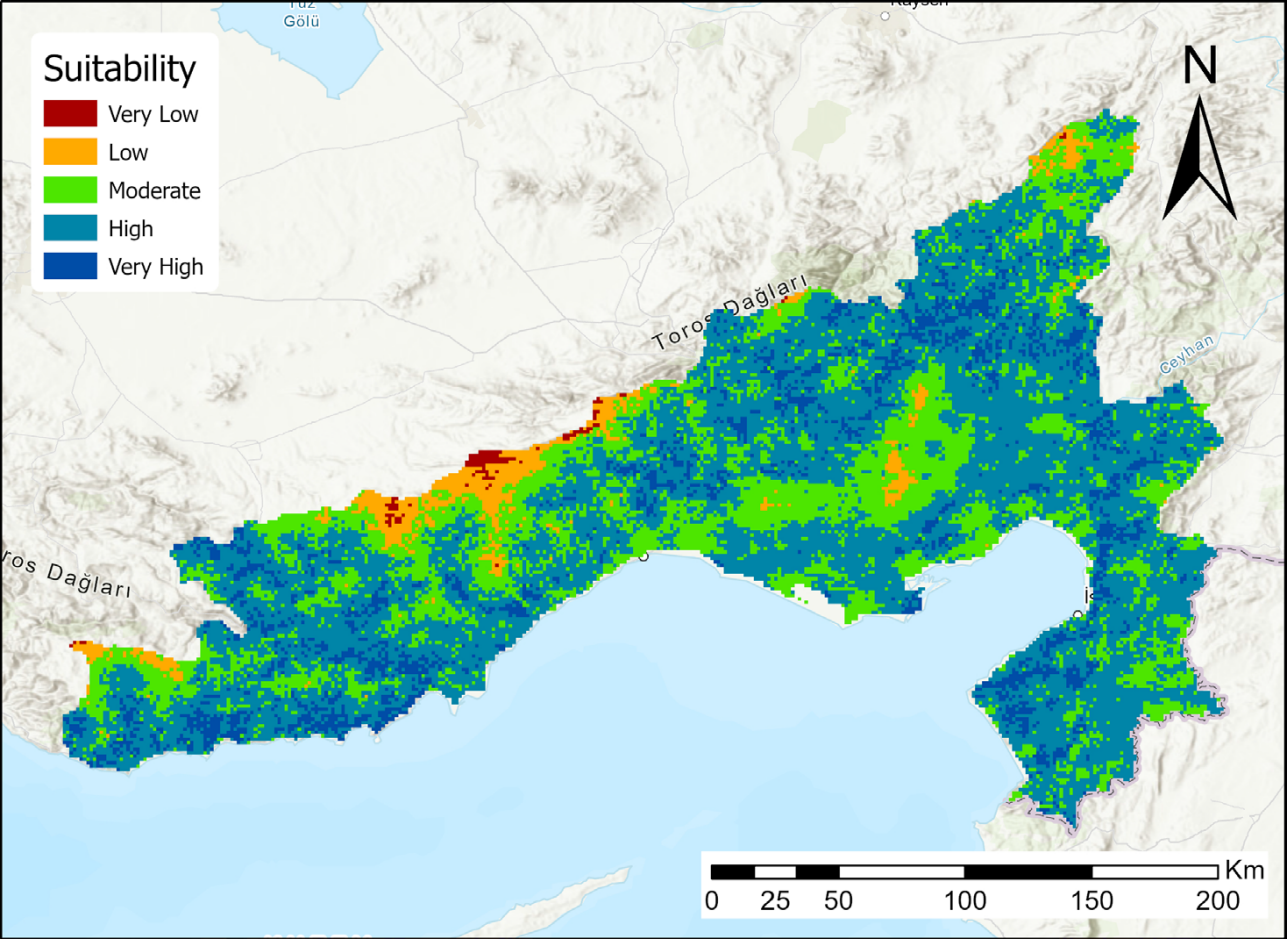
The suitability map of the spatial distribution of *Bufotes viridis* on the study area.

The final raster was reclassified into five ordinal categories to enable interpretation. Geospatial visualization of the suitability map (Figure 4) revealed that high suitability zones are generally located in areas characterized by (i) moderate elevations and low slope gradients, (ii) close proximity to freshwater sources, (iii) semi‐natural or forested land cover types, (iv) optimal temperature ranges and sufficient canopy cover. These environmental features are consistent with the ecological requirements of biodiversity‐rich habitats.

The results based on the distribution of suitability classes (Table 3) indicate that approximately 95.36% of the study area exhibits moderate, high, or very high ecological suitability, suggesting significant potential for conservation, biodiversity management, or low‐impact land use.

**TABLE 3:** Distribution of habitat suitability classes for *Bufotes viridis* across the study area.

Suitability class	Count of pixels	Percentage (%)	Area (km^2^)
Very low	125	0.47	125
Low	1104	4.17	1104
Moderate	7557	28.52	7557
High	15,077	56.91	15,077
Very high	2630	9.93	2630

### 3.3 Field‐Based Overlay Validation

**FIGURE 5:**
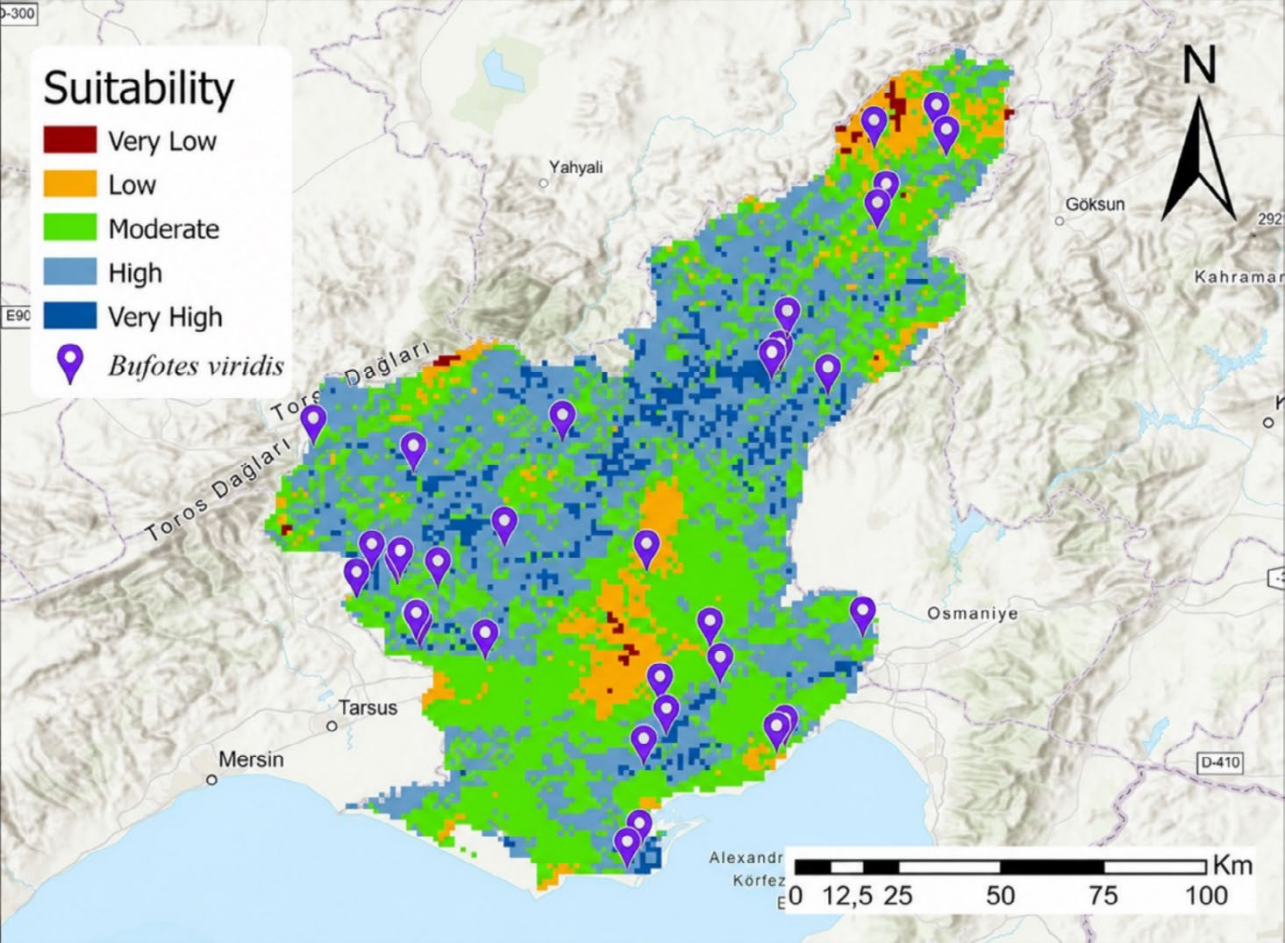
Overlap between the suitability map and the geographical distribution of *Bufotes viridis* in Adana Province, Türkiye.

To assess the ecological validity of the model outputs, 33 georeferenced field sampling locations of *B. viridis* from Adana Province were superimposed onto the final suitability map (Figure 5).

The overlay analysis was performed to obtain quantitative validation results (Table 4) by comparing the predicted suitability map (Figure 5) with the known distribution records of *B. viridis* in Adana Province (Altunışık et al. 2021; Sarıkaya et al. 2017).

**TABLE 4:** Distribution of habitat suitability classes and field occurrence records in Adana Province, Türkiye.

Suitability class	Count of pixels	Area (km^2^)	Frequency	Percentage (%)
Very low	55	55	0	0
Low	830	830	2	6
Moderate	4106	4106	12	36
High	3947	3947	15	46
Very high	620	620	4	12

In Table 4, Count of Pixels represents the number of raster cells (1 km^2^ each) associated with each suitability class. Frequency indicates the number of *B. viridis* occurrence records falling within each habitat suitability class.

The results based on the distribution of validation of the spatial distribution of *B. viridis* suitability classes (Table 4) indicate that approximately 94% in Adana Province in Türkiye exhibits moderate, high, or very high ecological suitability, suggesting significant potential for conservation, biodiversity management, or low‐impact land use.

## 4 Discussion

This study integrated a multi‐criteria decision‐making approach with GIS‐based spatial analysis to identify ecologically suitable areas across a heterogeneous Mediterranean landscape. The use of the AHP enabled the incorporation of expert knowledge to weight critical environmental factors, while field validation with georeferenced sampling points provided empirical support for the model's credibility.

### 4.1 Evaluation of Model Performance

The overlay of 33 field sampling points with the final suitability map showed that most occurrence records were located within areas classified as moderate, high, or very high suitability. Because these suitability classes collectively occupy a large proportion of the landscape, this result alone should not be considered a strong independent measure of model performance. Nevertheless, the spatial correspondence between occurrence records and environmentally favorable areas supports the ecological plausibility of the model and is consistent with the known habitat requirements of *B. viridis* . The high influence assigned to proximity to freshwater sources (weight = 0.352) and temperature (weight = 0.186) aligns well with ecological theory, where water availability and thermal conditions are known to shape habitat suitability and species distribution in Mediterranean ecosystems.

The spatial suitability model results are consistent with the known ecology of *B. viridis* (Altunışık et al. 2021). High suitability zones correspond to open habitats with moderate elevation, proximity to water bodies, and favorable thermal conditions. This aligns with field observations showing that the species readily occupies disturbed or semi‐natural habitats such as fallow fields, ruderal areas, and even construction sites (Landler et al. 2023). In contrast, densely forested landscapes and urban centers represent ecological barriers, corroborating previous findings of negative correlations between toad presence and dense vegetation or heavy urbanization (Landler et al. 2023; Sillero et al. 2014). Interestingly, our model also highlights saline and disturbed aquatic habitats as suitable zones, which reflects the species' well‐documented tolerance to salinity and ability to colonize novel or ephemeral aquatic habitats (Kuzmin 1999). This ecological flexibility may partly explain the species' success in anthropogenically altered landscapes, as reported in several European and Anatolian studies (Altunışık et al. 2021; Vargová et al. 2023).

### 4.2 Potential Threats to Habitat Suitability

While *B. viridis* shows ecological flexibility, this plasticity does not render populations immune to global and local threats. Climate change is expected to alter rainfall regimes and hydroperiods patterns, reducing the availability of ephemeral breeding sites and potentially shifting breeding phenology (Altunışık 2019; Altunışık et al. 2021). Extended drought periods may drastically reduce larval survival by shortening hydroperiods. Habitat fragmentation associated with urban expansion and transportation infrastructure can impede dispersal and gene flow. Although suitable habitats are widespread across the study area, local barriers such as major roads, dense urban areas, and the degradation of breeding wetlands may still limit movement at finer spatial scales and reduce connectivity among local populations (Vargová et al. 2023). This pattern is concerning because fragmentation increases the vulnerability of small, isolated populations to demographic stochasticity and local extinctions. Similar distributional shifts under projected climate change have been reported in other amphibians of the Mediterranean and North Africa, where altered precipitation regimes and temperature increases may drastically reduce habitat suitability (Kalboussi and Achour 2024; Pottier et al. 2025; Sun et al. 2025). This suggests that our predictions for *B. viridis* should be considered within the broader context of climate‐driven range contractions in amphibians. Recent studies also emphasize that land‐use and land‐cover changes significantly reshape amphibian distributions at regional scales, highlighting the importance of integrating LULC scenarios into future modeling efforts (Liu et al. 2024; Yang et al. 2025).

Emerging pathogens (e.g., *Batrachochytrium dendrobatidis* and ranaviruses) and pollutant loads in breeding waters represent additional, synergistic threats that may interact with climatic stressors to exacerbate declines; although pathogen data were not directly incorporated into this study, future risk assessments should consider disease exposure and water quality as modifiers of habitat suitability. Importantly, the high proportion of suitable habitat predicted by the model should not be interpreted as evidence that conservation measures are unnecessary. Habitat suitability reflects the presence of environmental conditions associated with species occurrence, but does not directly measure habitat quality, breeding success, population density, or long‐term persistence. In widespread and ecologically flexible species such as *B. viridis* , population declines may occur even within climatically suitable landscapes if breeding sites are degraded, hydroperiods are altered, or aquatic habitats become polluted.

### 4.3 Conservation Implications

The model outputs provide useful insights for conservation planning. Given that most of the study area was classified as suitable for *B. viridis* , habitat availability does not appear to be a major limiting factor at the regional scale. Instead, conservation efforts should focus on maintaining the quality of existing habitats, particularly breeding sites associated with freshwater resources, which emerged as the most influential predictor of habitat suitability. Protecting and, where possible, restoring ephemeral wetlands and shallow ponds‐especially within agricultural and peri‐urban landscapes—would help sustain breeding opportunities and population persistence. In addition, reducing pressures such as water pollution, wetland degradation, and road mortality may provide greater conservation benefits than large‐scale habitat expansion or connectivity interventions. Urban planning should also recognize the ecological value of semi‐natural open spaces and temporary water bodies. The incorporation of biodiversity‐friendly features, such as rain gardens and retained depressions, may enhance breeding opportunities within human‐modified landscapes. Finally, future studies integrating climate‐change projections with habitat suitability models would improve our understanding of potential range shifts and support the development of adaptive conservation strategies.

Although AHP provided a transparent and expert‐informed weighting framework, the approach remains partly subjective; complementing AHP with data‐driven modeling approaches (e.g., ensemble SDMs, machine learning) and testing sensitivity of results to alternative weighting schemes would strengthen inference. Our current validation with field points is promising, but expanding validation across more provinces and incorporating temporal (seasonal/interannual) data would better capture spatiotemporal variability in suitability. Besides, complementary approaches such as MaxEnt, ensemble species distribution models, or machine learning techniques (Cushman et al. 2024; Jamali et al. 2024) could be integrated with AHP to reduce subjectivity and strengthen predictive accuracy.

In addition, integrating climate‐change projections and hydrological models into suitability assessments is a priority: forecasting shifts in breeding‐site availability and thermal regimes will help identify refugia and areas likely to remain suitable under future scenarios. Finally, targeted field studies that link demographic rates (survival, fecundity) to predicted suitability classes would allow direct translation of suitability maps into population viability metrics.

Beyond ecological applications, the outputs of our suitability model can guide policymakers and local authorities in sustainable land‐use planning, particularly in rapidly urbanizing Mediterranean regions where biodiversity is increasingly threatened.

## 5 Conclusion

This study demonstrates the effectiveness of integrating multi‐criteria decision‐making (AHP) with GIS‐based spatial analysis to identify ecologically suitable areas across a heterogeneous landscape. By leveraging open‐source environmental data and expert‐derived weights, a composite suitability map was developed and validated using field sampling locations. The results highlight the dominant influence of proximity to freshwater sources, temperature, and altitude in determining ecological suitability across the study region. The high degree of spatial agreement between modeled suitability zones and real‐world field observations—particularly in Adana Province‐supports the robustness and applicability of the proposed approach. This integrative framework offers a scalable and adaptable model for environmental planning, biodiversity conservation, and site prioritization, especially in regions where field data are limited but spatial heterogeneity is high. The reproducibility and transparency of the methodology ensure its usability by both researchers and regional planners. Future research should expand the validation process across multiple ecological zones and explore hybrid approaches that combine expert knowledge with data‐driven machine learning models to enhance spatial prediction accuracy.

## Author Contributions

**Ali Erdem Özçelik:** conceptualization (equal), investigation (equal), methodology (equal), supervision (equal), validation (equal), writing – review and editing (equal). **Rıdvan Ertuğrul Yıldırım:** data curation (equal), methodology (equal), validation (equal), visualization (equal), writing – original draft (equal), writing – review and editing (equal). **Abdullah Altunışık:** conceptualization (equal), investigation (equal), supervision (equal), writing – original draft (equal), writing – review and editing (equal).

## Funding

The authors have nothing to report.

## Ethics Statement

The authors have nothing to report.

## Consent

The authors have nothing to report.

## Conflicts of Interest

The authors declare no conflicts of interest.

## Data Availability

The study relies entirely on publicly available open‐source geospatial datasets. The specific data sources, variables, and repositories used for the multi‐scale habitat suitability modeling are structured as: Topographic Data: Altitude, Slope, and Aspect layers were derived from the USGS Shuttle Radar Topography Mission (SRTM) digital elevation model (available at https://earthexplorer.usgs.gov/). Bioclimatic Data: Temperature variables were obtained from the WorldClim 2.1 database (available at https://www.worldclim.org/). Vegetation Data: Canopy cover was acquired from the ESRI Living Atlas of the World (available at https://livingatlas.arcgis.com/). Land Cover Data: Land Cover types were sourced from the CORINE Land Cover 2018 (CLC 2018) dataset via the Copernicus Land Monitoring Service (available at https://land.copernicus.eu/). Infrastructure & Hydrological Data: Vector layers for Distance to Roads, Distance to City Centers, and Distance to Fresh Water Sources were extracted and processed from OpenStreetMap (OSM) data (available at https://www.openstreetmap.org/). The complete Python‐based geospatial workflow (utilizing ArcGIS Pro ArcPy), including data preprocessing, factor weighting, and final habitat suitability validation, has been made publicly available to ensure full reproducibility. The code repository is hosted on GitHub (https://github.com/reyildirim/Bufotes_Habitat_Suitability) and is permanently archived on Zenodo at https://doi.org/10.5281/zenodo.20843557.
